# Supplementary material for: Modeling glioblastoma heterogeneity as a dynamic network of cell states
Source: Mol Syst Biol. 2021 Sep 16;17(9):e10105. doi: 10.15252/msb.202010105 (PMC8444284; doi:10.15252/msb.202010105)
Supplement: Supplementary file 6 — Source Data for Figure 5 [file MSB-17-e10105-s004.zip › Figure5A_sourcedata/GSEA_3071/hallmarks_stateIV.GseaPreranked.1623742551702/HALLMARK_NOTCH_SIGNALING.html]

Details for gene set HALLMARK\_NOTCH\_SIGNALING[GSEA]

|  || Dataset | state53071 |
| Phenotype | NoPhenotypeAvailable |
| Upregulated in class | na\_pos |
| GeneSet | HALLMARK\_NOTCH\_SIGNALING |
| Enrichment Score (ES) | 0.12090821 |
| Normalized Enrichment Score (NES) | 0.41023964 |
| Nominal p-value | 0.9954023 |
| FDR q-value | 0.9977878 |
| FWER p-Value | 1.0 |
Table: GSEA Results Summary

  

Fig 1: Enrichment plot: HALLMARK\_NOTCH\_SIGNALING      
 Profile of the Running ES Score & Positions of GeneSet Members on the Rank Ordered List

  

| PROBE | GENE SYMBOL | GENE\_TITLE | RANK IN GENE LIST | RANK METRIC SCORE | RUNNING ES | CORE ENRICHMENT || 1 | MAML2 |  |  | 470 | 0.841 | 0.0173 | Yes |
| 2 | NOTCH1 |  |  | 666 | 0.678 | 0.0661 | Yes |
| 3 | ST3GAL6 |  |  | 785 | 0.610 | 0.1209 | Yes |
| 4 | PRKCA |  |  | 1214 | 0.461 | 0.0981 | No |
| 5 | DLL1 |  |  | 1463 | 0.399 | 0.1016 | No |
| 6 | SAP30 |  |  | 1914 | 0.311 | 0.0557 | No |
| 7 | FZD5 |  |  | 2089 | 0.285 | 0.0588 | No |
| 8 | SKP1 |  |  | 2150 | 0.276 | 0.0823 | No |
| 9 | CUL1 |  |  | 2617 | -0.285 | 0.0299 | No |
| 10 | RBX1 |  |  | 2951 | -0.325 | 0.0080 | No |
| 11 | DTX2 |  |  | 3322 | -0.379 | -0.0142 | No |
| 12 | TCF7L2 |  |  | 3497 | -0.409 | 0.0046 | No |
| 13 | NOTCH2 |  |  | 3853 | -0.477 | -0.0023 | No |
| 14 | LFNG |  |  | 3991 | -0.509 | 0.0361 | No |
| 15 | JAG1 |  |  | 4168 | -0.559 | 0.0735 | No |
| 16 | NOTCH3 |  |  | 5003 | -1.098 | 0.0544 | No |
Table: GSEA details [plain text format]

  

Fig 2: HALLMARK\_NOTCH\_SIGNALING: Random ES distribution      
 Gene set null distribution of ES for **HALLMARK\_NOTCH\_SIGNALING**
